# Bamboo Nanocellulose/Montmorillonite Nanosheets/Polyethyleneimine Gel Adsorbent for Methylene Blue and Cu(II) Removal from Aqueous Solutions

**DOI:** 10.3390/gels9010040

**Published:** 2023-01-04

**Authors:** Xuelun Zhang, Feng Li, Xiyu Zhao, Jiwen Cao, Shuai Liu, You Zhang, Zihui Yuan, Xiaobo Huang, Cornelis F. De Hoop, Xiaopeng Peng, Xingyan Huang

**Affiliations:** 1 College of Forestry, Sichuan Agricultural University, Chengdu 611130, China; 2 Research Institute of Characteristic Flowers and Trees, Chengdu Agricultural College, Chengdu 611130, China; 3 State Key Laboratory of Tree Genetics and Breeding, Key Laboratory of Tree Breeding and Cultivation of the National Forestry and Grassland Administration, Research Institute of Forestry, Chinese Academy of Forestry, Beijing 100091, China; 4 Key Laboratory of Pulp and Paper Science & Technology of Ministry of Education, Qilu University of Technology (Shandong Academy of Sciences), Jinan 250353, China; 5 National Forestry and Grassland Administration Key Laboratory of Plant Fiber Functional Materials, Fuzhou 350108, China; 6 School of Renewable Natural Resources, Louisiana State University Agricultural Center, Baton Rouge, LA 70803, USA; 7 Wood Industry and Furniture Engineering Key Laboratory of Sichuan Provincial Department of Education, Chengdu 611130, China

**Keywords:** gel, adsorption, nanocellulose, montmorillonite, methylene blue, heavy metal

## Abstract

In recent years, the scarcity of pure water resources has received a lot of attention from society because of the increasing amount of pollution from industrial waste. It is very important to use low-cost adsorbents with high-adsorption performance to reduce water pollution. In this work, a gel adsorbent with a high-adsorption performance on methylene blue (MB) and Cu(II) was prepared from bamboo nanocellulose (BCNF) (derived from waste bamboo paper) and montmorillonite nanosheet (MMTNS) cross-linked by polyethyleneimine (PEI). The resulting gel adsorbent was characterized by Fourier transform infrared spectroscopy (FTIR), field emission scanning electron microscopy (SEM), X-ray photoelectron spectroscopic (XPS), etc. The results indicated that the MB and Cu(II) adsorption capacities of the resulting gel adsorbent increased with the solution pH, contact time, initial concentration, and temperature before equilibrium. The adsorption processes of MB and Cu(II) fitted well with the fractal-like pseudo-second-order model. The maximal adsorption capacities on MB and Cu(II) calculated by the Sips model were 361.9 and 254.6 mg/g, respectively. The removal of MB and Cu(II) from aqueous solutions mainly included electrostatic attraction, ion exchange, hydrogen bonding interaction, etc. These results suggest that the resulting gel adsorbent is an ideal material for the removal of MB and Cu(II) from aqueous solutions.

## 1. Introduction

In recent years, many pollutants, such as nitrates [1,2], phosphorus [3], antibiotics [4], dyes [5], and heavy metals [6], have been discharged into the water as a result of rapid industrial development and human activities. Dyes and heavy metals are two of the most typical pollutants in industrial wastewater. They have serious impacts on human living environments [7,8]. The dyes in the water bodies can absorb light and hinder the penetration of light, thereby reducing the photosynthetic activities of aquatic plants and microorganisms, and inhibiting their growth [9]. Methylene blue (MB), a cationic dye, is widely used in textile, paper, coating, and printing [10,11]. The residual MB in wastewater has a serious influence on the environment and human health due to its non-biodegradable feature [12]. Cu(II), a common used heavy metal, is produced during copper smelting, processing, and electroplating [13]. Due to its toxicity and non-degradability in water, it could gradually accumulate into food chains, endangering human health [14]. As a result, Cu(II) is one of the priority pollutants classified by the US EPA [15]. Therefore, MB and Cu(II) in wastewater must be eliminated before being discharged into the environment.

The removal methods of MB and Cu(II) include ion exchange [16], chemical precipitation [17], electrodialysis [18], flocculation [19], membrane technology [20], adsorption [21], electrodialysis, etc. Chemical precipitation and flocculation are simple to operate, but they usually produce sludge and are difficult to remove; membrane technology and ion exchange introduce new chemicals; electrodialysis is also difficult to put into practical use given the high costs involved [22]. Adsorption is one of the most useful strategies in wastewater treatment because of its convenience, effectiveness, low cost, and no harmful products [23]. Many adsorbent materials, such as activated carbon, carbon nanotube, graphene, and metal-organic framework, have been applied to remove dyes and heavy metals in wastewater [24,25,26,27]. It is worth noting that some natural adsorbents, such as loofah sponges and sugarcane bagasse, can also remove pollution in wastewater, even though their adsorption capacities are not satisfactory [28,29]. The development of a natural, cheap, efficient, and biodegradable adsorbent is still a major concern in the study of wastewater treatment [30].

Cellulose is a naturally acquirable material with good biodegradability and renewability [31]. Nanoscale cellulose, i.e., nanocellulose (CNF) and cellulose nanocrystal (CNC) were developed from cellulose-containing materials [32]. The high aspect ratio, abundant hydroxyl groups, and structural flexibility of CNF are beneficial to removing dyes and heavy metal ions from wastewater. For example, CNF gel absorbent produced from TEMPO-oxidized CNF has a good adsorption effect on cationic dye (malachite green) [33]. Moreover, the adsorption efficiency of CNF gel toward Cu(II) could be enhanced via polyethyleneimine (PEI) [34].

Montmorillonite (MMT) is a clay mineral in nature. It has been demonstrated that MMT could be used as a reinforcing agent in the cellulose framework [35]. MMT has a three-layer sheet structure, consisting of a middle layer of aluminum oxide octahedron (Al^3+^) between two layers of silicon-oxygen tetrahedrons (Si^4+^) [36]. The Al^3+^ and Si^4+^ in MMT will be replaced by other cations with lower charges, resulting in a negatively charged MMT [37]. To eliminate the charge imbalance in the crystal lattice, a large number of exchangeable cations, such as Na^+^ and Ca^2+^, will be generated between layers [38]. The overall negative charge and cation exchange in MMT will contribute to its good adsorption performance on cationic pollutants in wastewater [5]. In addition, the lattice structure of MMT is maintained by weak electrostatic force and van der Waals force. It is easy to exfoliate MMT into nanosheets [39]. It will boost its surface areas, resulting in the full exposure of adsorption sites and functional groups [40,41]. Therefore, MMT nanosheets (MMTNS) have great potential to be used as adsorbents.

The application of gel in the removal of dyes and heavy metal ions from wastewater has received a lot of attention [16,42]. The gel structure is built from hydrophilic groups, for example, –OH, –COOH, –NH_2_, –CONH_2_, and –SO_3_H, or hydrated polymer networks under aqueous conditions [43]. Many materials have been used to prepare gels, such as chitosan, alginate, and cellulose. However, these biopolymers have many drawbacks, such as low stability and limited potential to remove dye molecules. Cross-linking is the most critical strategy to prepare the gel with a stable 3D structure for improving its physical and mechanical properties [44,45]. For example, gel beads prepared from sodium alginate and carboxymethyl cellulose by blending and cross-linking have adsorption capacities for lead ions due to their hydroxyl and carboxyl groups [46].

Polyethyleneimine (PEI) has a large number of amino groups that have good adsorption capacities in heavy metal ions and they are able to form cross-linking sites with other functional groups to enhance the structure stability [47].In this study, PEI with abundant amino groups was used as a cross-linking agent to reinforce the connection between bamboo nanocellulose and MMTNS for producing a stable gel adsorbent. Similar work on CNF/MMT/PEI had similar raw materials to ours [35]. The authors focused on the adsorption of anionic dye through amino groups from PEI, while MMT was overall negatively charged, it could be easily exfoliated into nanosheets to increase its surface areas and adsorption sites for positively charged cationic dyes.

Thereby, the performance of the resulting gel adsorbent in this work to remove MB and Cu(II) from the aqueous solutions was investigated. The novelty of this work was the utilization of waste bamboo paper, montmorillonite, and polyethyleneimine as raw materials, for their low costs and great potential adsorption abilities after combining, and to prepare bio-based gel adsorbents, which had good adsorption capacities for MB and Cu(II). The adsorption processes and maximum adsorption capacities were determined by a kinetic study and adsorption isotherm, respectively. Furthermore, SEM and XPS analyses were carried out to study the adsorption mechanism.

## 2. Results and Discussion

### 2.1. Characterization of the BMP Gel Adsorbent

#### 2.1.1. FTIR Spectra

**Figure 1:**
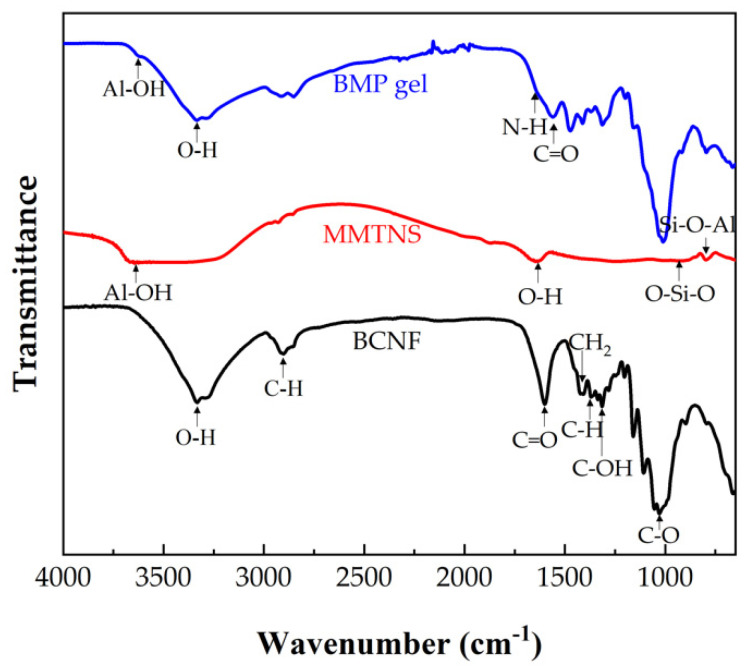
FTIR spectra of BCNF, MMTNS, and BMP gel adsorbent.

**Figure 2:**
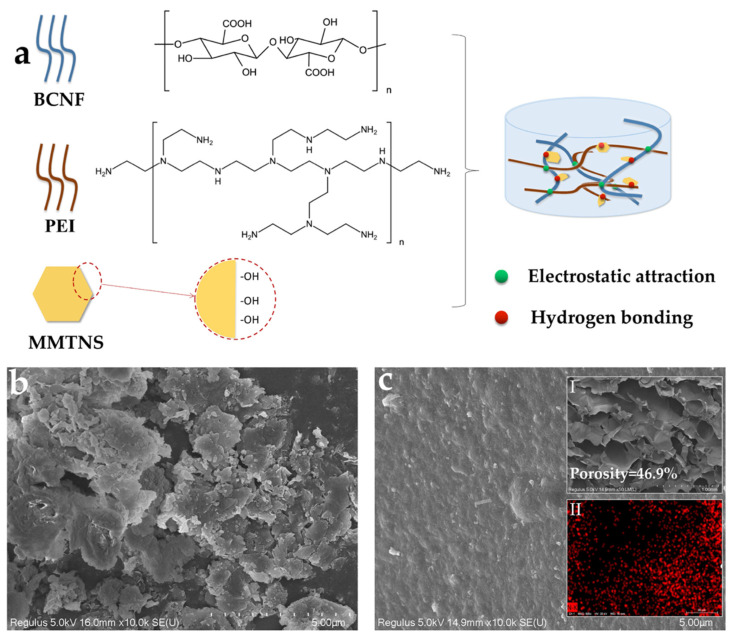
Proposed preparation mechanism of the BMP gel adsorbent (**a**); SEM images of MMTNS (**b**) and the BMP gel adsorbent (**c**).

The FTIR spectra of BCNF, MMTNS, and BMP gel are shown in Figure 1. The remarkable BCNF bands at 3334 and 1601 cm^−1^ corresponded to O–H stretching, and C=O stretching, respectively [48]. The bands observed at approximately 2903 cm^−1^ wereattributed to the symmetrical stretching vibration of the C–H bond [49]. The band at 1431 cm^−1^ corresponded to CH_2_ deformation; 1375–1315 cm^−1^ was assigned to C-H and C-OH deformation, and 1160–896 cm^−1^ corresponded to the C–O-stretched backbone vibrations and glycosidic linkages between sugar units [50,51]. A strong band at 3633 cm^−1^, assigned to Al–OH vibration, was observed from MMTNS [52]. The main bands at 1640, 930, and 795 cm^−1^ corresponded to the stretching vibration of the hydroxyl groups, O–Si-O stretching, and the Si–O–Al stretching in MMTNS, respectively [53,54]. Moreover, it was found that the band of hydrogen bonding in the BMP gel was well retained at 3334 cm^−1^. These findings indicated that strong hydrogen bonding interactions were formed between BCNF and MMTNS [55]. Meanwhile, a secondary amide shoulder from PEI was observed at 1649 cm^−1^ of the BMP gel adsorbent, indicating that PEI was involved successfully [56]. Moreover, a strong stretching vibration band of carboxylic acid anion was found at 1560 cm^−1^ on the IR spectrum of the BMP gel adsorbent [57]. These findings suggested the formation of a cross-linked structure between –COOH (BCNF) and -NH_2_ (PEI) through an electrostatic attraction (charged salt groups formed–NH_3_(+)/–NH_2_–(+) and (−)OOC– interactions). The proposed preparation mechanism of the BCNF/MMTNS/PEI gel adsorbent is shown in Figure 2a.

#### 2.1.2. SEM

The surface morphology and structure of the MMTNS and BMP gel adsorbent are presented in Figure 2. There were many nanosheets on the surface of MMTNS (Figure 2b), suggesting that MMT was successfully exfoliated by ultrasonic separation. It can be observed from Figure 2c that the gel adsorbent had a three-dimensional layered structure with a large interlayer space. The porosity of the BMP gel adsorbent was calculated to be 46.9% using Image J. The resulting gel adsorbent prepared from BCNF and MMTNS had a porous structure. It could provide channels for the adsorbate to pass through [58]. The cell wall of the BMP gel adsorbent (Figure 2c) showed a dense, smooth, and non-porous surface, suggesting that BCNF and MMTNS dispersed uniformly and connected tightly after cross-linking. It is worth noting that N elements were observed on the EDS image of the BMP gel, indicating that PEI was successfully involved.

### 2.2. MB and Cu(II) Adsorption

#### 2.2.1. Effect of Initial pH

**Figure 3:**
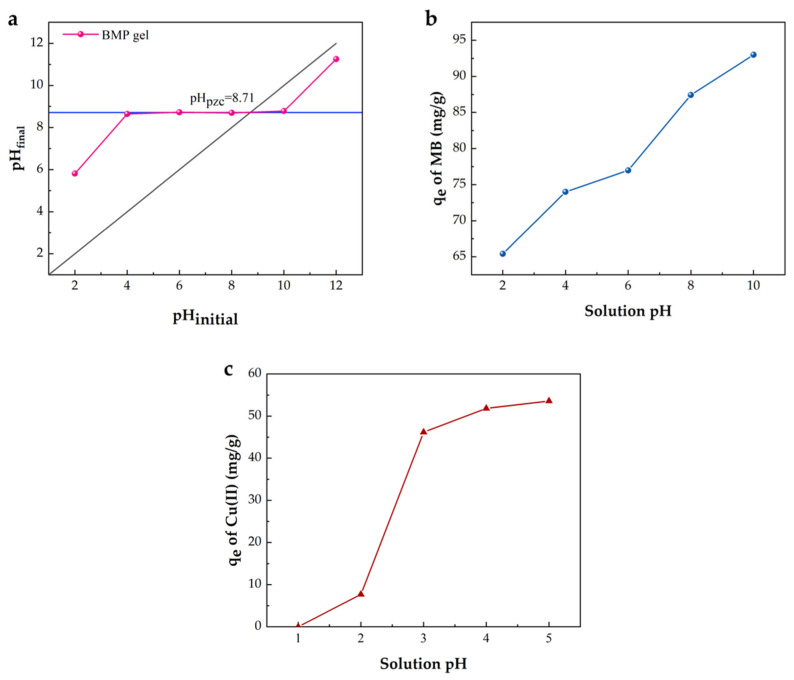
pH_pzc_ of the BMP gel adsorbent (**a**); effect of the solution pH on the adsorption of MB (**b**) and Cu(II) (**c**) (C_0_ = 100 mg/L, t = 24 h, T = 25 °C).

The solution pH is an important factor affecting the adsorption process [59]. The point of zero charge (pH_pzc_) is the pH at which the positive and negative charges are balanced (dissociation into the liquid by H^+^ and OH^−^ ions) [60]. Thus, pH_pzc_ is a useful measurement for assessing the surface acidity of the BMP gel and characterizing functional groups on its surface. The pH_pzc_ of the BMP gel was 8.71, as shown in Figure 3a. It indicated that the surface of the BMP gel was negatively charged at the pH solution> 8.71, and vice versa.

MB is a cationic dye in a wide pH range from 0 to 14. It was observed that the adsorption capacity of MB increased sharply with an increase of the solution pH, and the maximum adsorption capacity was 93.0 mg/g at pH = 10 (Figure 3b). MB could be easily adsorbed on the external surface and pores of the resulting gel adsorbent that is larger than its molecular size [50]. Moreover, MB is either mono- (HMB^2+^) or di-protonated (H_2_MB^3+^) at acidic pH conditions [61]. The low MB adsorption at pH < pH_pzc_ was due to the competition between H^+^ and MB. With the increasing solution pH, most of the carboxyl groups in the BMP gel adsorbent were ionized into carboxylate anions (–COO^−^); thus, the strong electrostatic adsorption between the negative surface charge and the cationic dye molecule increased [62]. Furthermore, the hydrogen bond interaction between the imine group (RCH = NR) of the MB molecule and the –OH group of gel adsorbent could enhance the adsorption of MB [63].

Cu(II) in solution is easily converted into copper hydroxide precipitation when pH is above 5. Therefore, the effect of pH on the adsorption of Cu(II) was studied in acid environments (from 1 to 5) at 25 °C in this work (Figure 3c). The maximum adsorption capacity of Cu(II) on the BMP gel was 53.6 mg/g at pH = 5. The adsorption of Cu(II) improved with the increase of solution pH. The protonation of the amino groups on PEI was dominant (as pH ≤ 2). As a result, there was an electrostatic repulsion between the BMP gel adsorbent and Cu(II) [64]. As the solution pH increased, the electrostatic force between MMTNS and the original interlayer cation gradually strengthened and the competition from H^+^ tended to weaken [65]. Meanwhile, amino groups were deprotonated, thereby improving the adsorption capacity of Cu(II) [66].

#### 2.2.2. Adsorption Kinetics and Isotherms

**Figure 4:**
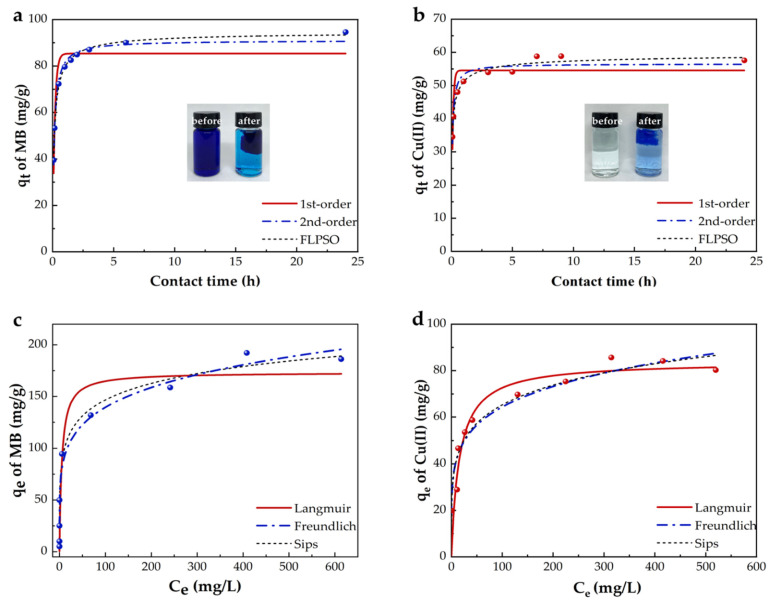
Kinetic models for MB adsorption (**a**) (pH = 10, C_0_ = 100 mg/L, T = 25 °C) and Cu(II) adsorption (**b**) (pH = 5, C_0_ = 100 mg/L, T = 25 °C); (**c**) isothermal models for MB adsorption (pH = 10, t = 24 h, T = 25 °C) and Cu(II) adsorption (**d**) (pH = 5, t = 24 h, T = 25 °C).

Figure 4 displays the adsorption kinetics and isotherms of MB and Cu(II) into the BMP gel adsorbent. Table 1 lists the fitting parameters. MB and Cu(II)were adsorbed to reach adsorption equilibrium within 6 and 10 h, respectively. The adsorption rate slowed down with time, and the adsorption capacity tended to be stable. It suggested that the adsorption rate was a time-dependent factor. The physical meaning of time dependence is that the reaction path changes with time [67]. It can be observed from Figure 4a,b that the adsorption processes of MB and Cu(II) were fitted better by the fractal-like pseudo-second-order model than the other kinetic models. The correlation coefficients (*R*_3_^2^) were 0.99 and 0.97, respectively. Moreover, the low reduced Chi-Sqr value also verified that the fractal-like pseudo-second-order model was the most suitable one to fit MB and Cu(II) adsorption processes [68].

The concentration dependence adsorptions of MB and Cu(II) by the BMP gel adsorbent are shown in Figure 4c,d. The obtained high correlation coefficient (*R*^2^) and low reduced Chi-Sqr values from these isothermal Langmuir, Freundlich, and Sips models are represented in Table 1. The *R*_6_^2^ values (MB: 0.97; Cu(II): 0.93) calculated from the Sips isotherm model were greater than the other models, indicating that the Sips isotherm better described the adsorptions of MB and Cu(II) by the adsorbent BMP gel than the other ones. This model indicated that the adsorption processes of MB and Cu(II) were followed by a combined model: monomolecular (at high concentration) and diffuse (at low concentration) [69]. The maximal adsorption capacities of MB and Cu(II) were 361.9 and 254.6 mg/g, respectively, calculated by the Sips isotherm model, and were higher than most of the previously reported works (Table 2). In addition, the adsorptions of MB and Cu(II) were fitted well by the Freundlich model. The n^−1^ in Freundlich indicates the advantage of the adsorption process. If n^−1^ < 1, the adsorption intensity is favorable over the entire range of the concentration studied. If n^−1^ > 1, it means that the adsorption capacity is desirable at a high concentration but much less so at a lower concentration [14]. The value of n^−1^ is 0.19 for both MB and Cu(II) (Table 1), indicating favorable adsorptions over the entire concentration ranges for both MB and Cu(II).

#### 2.2.3. Effect of Interfering Ions

**Figure 5:**
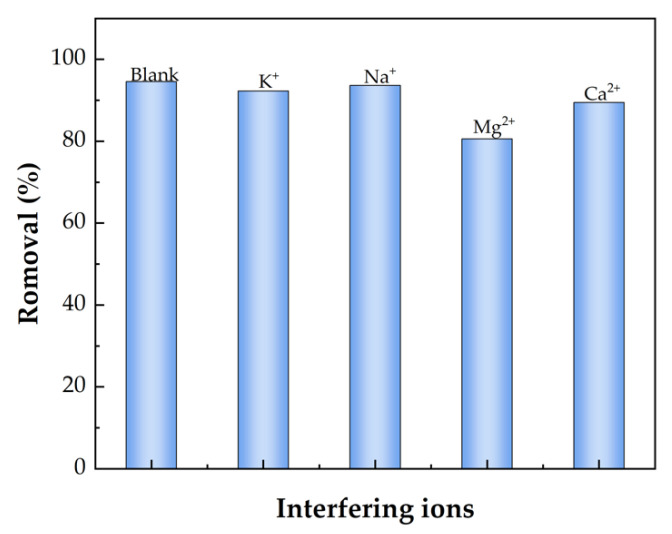
Effect of interfering ions on the MB adsorption by the BMP.

Actual wastewater contains several common ions, such as K^+^, Na^+^, Mg^2+^, Ca^2+^, etc. To check the effects of these ions, we studied the adsorption of MB into BMP in the presence of 100 mg/L of a dye solution with 10 mM aqueous solutions of salt. The results are shown in Figure 5. The adsorption of MB into BMP was 94.6% in the blank group. By adding these interfering ions, the adsorptions of MB slightly decreased in the order of Na^+^(93.7%) > K^+^(92.3%) > Ca^2+^ (89.5%)> Mg^2+^ (80.8%). The decrease in the removal was due to differences in the radii of the hydrated ions. Ions with smaller hydrodynamic radii can compete with larger-sized contaminants and are easily absorbed, resulting in a decrease in MB removal [72]. It is worth noting that BMP can effectively remove MB from aqueous media even in the presence of interfering ions.

#### 2.2.4. Adsorption Thermodynamics and Adsorbent Reusability

**Figure 6:**
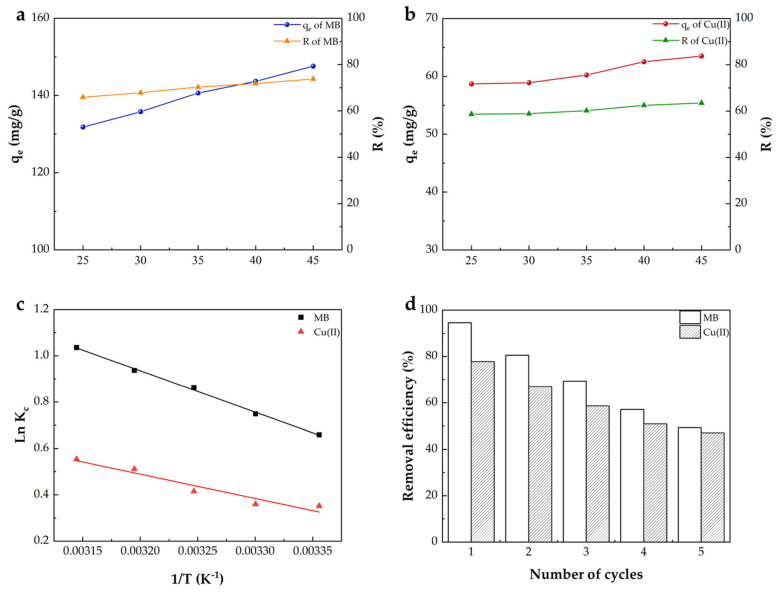
Effect of temperature on the adsorption of (**a**) MB (C_0_ = 200 mg/L; the pH solution = 10) and (**b**) Cu(II) (C_0_ = 100 mg/L; the pH solution = 5); the plot of LnKc versus 1/T (**c**); reusability of the BMP gel adsorbent for MB and Cu(II) (**d**).

As the temperature increased from 25 to 45 °C (Figure 6a,b), the adsorption capacities of MB and Cu(II) by the BMP gel adsorbent increased from 131.8 to 147.6 mg/g and from 58.7 to 63.5 mg/g, respectively. Meanwhile, the MB and Cu(II) removal efficiencies increased from 65.9% to 73.8% and from 58.7% to 63.5%, respectively, with increasing temperatures. The thermodynamic parameters of the MB and Cu(II) adsorptions by the BMP gel adsorbent are presented in Table 3. The positive Δ*H°* elucidated that the adsorption mechanism was endothermic, which implied that a large amount of heat was required to transfer dyes and metal ions from the aqueous phase to the solid phase. The negative value of Gibbs free energy (Δ*G°*) indicated that the adsorptions of MB and Cu(II) on the adsorbent were spontaneous [72,73]. The positive Δ*S°* values suggested an increase in the degrees of randomness at the interface between the solid and liquid during the adsorption of MB and Cu(II) on the resulting BMP gel adsorbent [53].

The regeneration of the adsorbent was also studied to provide a basis for its practical application. The reusability of the BMP gel adsorbent was tested by repeating five cycles of the adsorption–desorption process (Figure 6d). After five cycles, the removal efficiencies for MB and Cu(II) remained at 49.3% and 47.1%, respectively, indicating that they had acceptable reusability. The reduction of removal efficiency could be attributed to the incomplete desorption of MB and Cu(II) [74]. It is easy to remove the gel from the suspension for its solid shape, thereby, it has good potential to be used as an industrial adsorbent.

#### 2.2.5. Adsorption Mechanism Analysis

**Figure 7:**
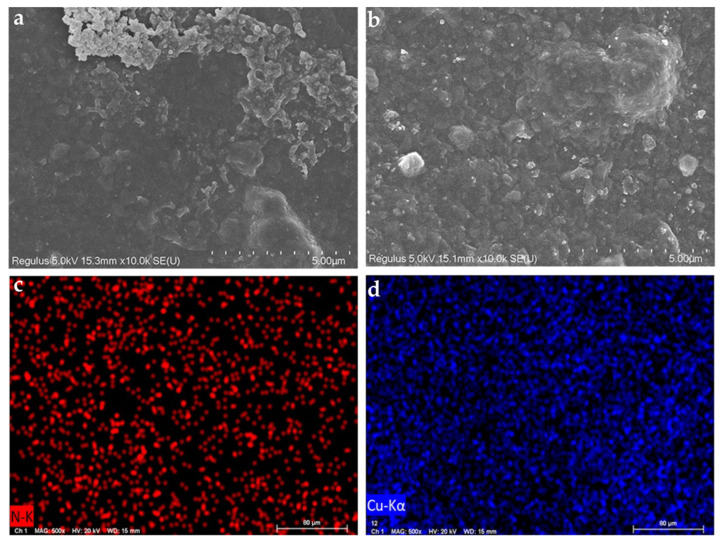
SEM images of the gel adsorbent after the adsorption of MB (**a**) and Cu(II) (**b**); EDS images of gel adsorbent after the adsorption of MB (**c**) and Cu (II) (**d**).

Figure 7 shows the SEM images of the gel adsorbent after the adsorptions of MB and Cu(II). Compared with the smooth surface before the adsorption (Figure 2c), the gel adsorbent surface became rougher after adsorbing MB and Cu(II) (Figure 5a,b). Furthermore, as shown in Figure 5d, the blue points (denoted Cu elements) are evenly distributed on the surface of the adsorbed BMP gel adsorbent, suggesting that Cu was evenly covered on the surface of the BMP gel adsorbent. These results indicate the successful adsorptions of MB and Cu(II), and the uniform distribution of the adsorption sites on the adsorbent. These SEM images suggest that the BMP gel adsorbent had great potential to be used as an adsorbent candidate.

**Figure 8:**
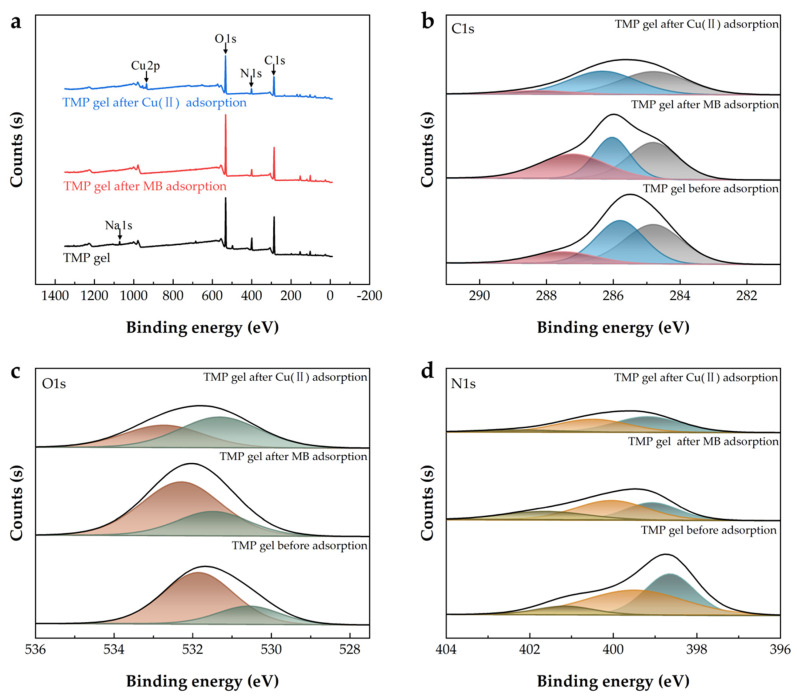
XPS spectra of the BMP gel adsorbent before and after adsorption on MB and Cu(II) (**a**); C1s region (**b**); O1s region (**c**); N1s region (**d**).

The chemical compositions of the BMP gel adsorbents before and after adsorption were characterized by XPS. As shown in Figure 8a, there was an obvious decrease of Na1s in the BMP gel adsorbent after MB and Cu(II) adsorptions. The wide scan spectra of pristine BMP gel adsorbent did not have any signal in the Cu2p region, but binding energy peaks at around 934.36 eV appeared after Cu(II) adsorption. These findings suggest that the exchangeable cations, Na^+^, existed in MMTNS [65]. In the C1s plot (Figure 8b), the binding energies of C–O and O–C=O were at 285.79 and 287.50 eV before adsorption, respectively [75]. They shifted to 286.03 and 287.20 eV after MB adsorption and shifted to 286.32 and 288.32 eV after Cu(II) adsorption, owing to the combination of groups, such as -OH and -COOH on the BMP gel adsorbent with MB or Cu(II) [76]. The O1s initial peak could be divided into two regions at 530.57 and 531.85 eV, being consistent with the oxygen of C=O and C–O (Figure 8c) [77]. They shifted to 531.48 and 532.28 eV after the MB adsorption and shifted to 531.31 and 532.73 eV after the Cu(II) adsorption, which suggested that oxygen atoms in carboxyl and hydroxyl groups were involved in the adsorption process [21]. The N1s spectra of the BMP gel adsorbent before adsorption had peaks at 398.64, 399.49, and 401.15 eV, which were assigned to -NH_2_, -NH-, and -NH_3_^+^, respectively (Figure 8d) [48,78]. After MB was adsorbed, the binding energy peaks of N1s shifted to 399.08, 400.04, and 401.67 eV. After Cu(II) was adsorbed, the binding energy peaks of N1s shifted to 399.18, 400.49, and 402.25 eV. Simultaneously, there was an obvious decrease in the N1s intensities. These findings confirmed that all three kinds (primary amine, secondary amine, and tertiary amine groups) of amino groups from PEI were involved in the removal of MB and Cu(II) [77].

**Figure 9:**
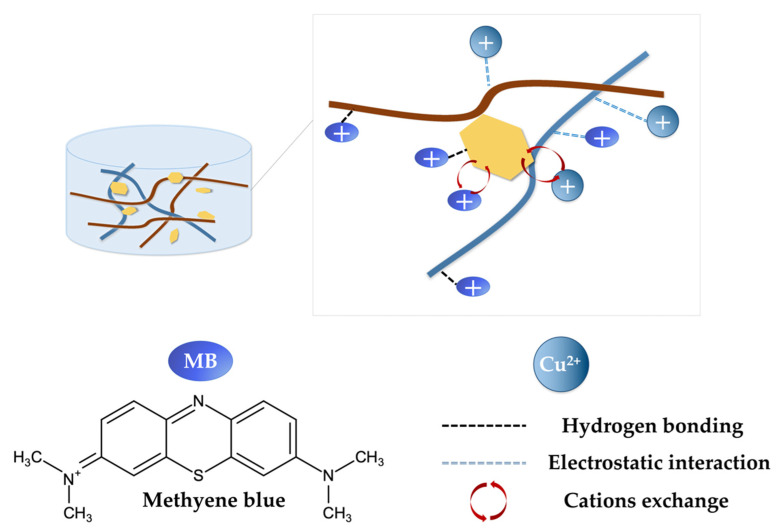
The proposed adsorption mechanism of MB and Cu(II) by the BMP gel adsorbent.

Under the alkaline condition, the cationic group of MB could be easily attracted by the deprotonated carboxyl group in the BMP gel adsorbent through the electrostatic interaction. A large number of hydroxyl groups in the BMP gel adsorbent could form hydrogen bonds with the imine groups (RCH=NR) of MB molecules, which also enhanced adsorption. In addition, the van der Waals force may play an important role in the MB adsorption process [79]. As for the adsorption of Cu(II), the partially ionized carboxyl groups in the BMP gel adsorbent could form electrostatic adsorption with Cu(II). The PEI grafted on the gel adsorbent had a large number of amino groups and could also be connected with Cu(II) to promote adsorption [34]. It is also worth noting that MB molecules and Cu(II) ions could exchange cations with ions between the MMTNS layers [16]. Comprehensively, BCNF was rich in hydroxyl and carboxyl groups, which help form a stable structure of the BMP gel adsorbent. It could adsorb positively charged MB and Cu(II). MMTNS could be used as a reinforcement agent for cellulose framework, and the cations between the MMTNS layers could exchange ions with MB and Cu(II). PEI, as a cross-linking agent with a large number of amino groups, could increase the structural stability and boost the removal efficiencies of MB and Cu(II). From the above, the proposed adsorption mechanisms of MB and Cu(II) by the BMP gel adsorbent are shown in Figure 9.

## 3. Conclusions

In this study, the BCNF/MMTNS/PEI (BMP) gel adsorbent was successfully prepared from bamboo nanocellulose and montmorillonite nanosheets, cross-linked by polyethyleneimine, and used as an adsorbent to remove the cationic dye, methylene blue (MB), heavy metal, and Cu(II) from aqueous solutions. The FTIR and SEM results showed that the resulting gel adsorbent had abundant hydroxyl, carboxyl, and amino groups, as well as a porous structure. The kinetics study of adsorption processes on MB and Cu(II) was well-fitted by a fractal-like pseudo-second-order model. According to the Sips isotherm model, the calculated maximum adsorption capacities of MB and Cu(II) were 361.9 mg/g and 254.6 mg/g, respectively. The adsorption mechanisms mainly included electrostatic attraction, ion exchange, hydrogen bond interactions, etc. These results suggest that the adsorbent had great potential to be used to remove MB and Cu(II) from aqueous solutions.

## 4. Materials and Methods

### 4.1. Materials

The waste bamboo paper was collected from the laboratory. Montmorillonite (MMT) was bought from Zhejiang Fenghong New Materials co., Ltd. (Huzhou, China). Methylene blue (MB), copper sulfate pentahydrate, and 2,2,6,6-Tetramethylpiperidine (TEMPO) were obtained from Sinopharm Chemical Regent Co., Ltd. (Shanghai, China). Polyethyleneimine (PEI, 50% aqueous solution) was purchased from Aladdin Reagent Co., Ltd. (Shanghai, China). Sodium hypochlorite (NaClO), glacial acetic acid (CH_3_COOH), sodium bromide (NaBr), sodium hydroxide (NaOH), and hydrochloric acid (HCl) were obtained from Chengdu Kelong Chemical Co., Ltd. (Chengdu, China). All chemicals were of analytical grade and used without further purification.

### 4.2. Preparation of Bamboo Nanocellulose (BCNF)

BCNF was prepared from waste bamboo paper in accordance with the previously reported methods [80]. Briefly, cellulose was obtained from the waste bamboo paper after bleaching and alkali treatment. Then 10 g of cellulose was mixed with 1000 mL of deionized water, 0.36 g of TEMPO, and 37.5 mL of NaClO. Then, the pH was adjusted to 10 by HCI and NaOH. Next, the suspension was treated with an ultrasonic homogenizer (Scientz, China) for 1 h. Finally, it was freeze-dried to obtain BCNF.

### 4.3. Preparation of MMTNS

The 5 wt% of MMT suspension was centrifuged at a speed of 1000 r/min for 2 min to remove large particles. Then 7 wt% of MMT was exfoliated by an ultrasonic processor (Scientz, China) at 400 w of power for 15 min. Finally, the exfoliated MMTNS sample was dried at 60 °C.

### 4.4. Preparation of BCNF/MMTNS/PEI (BMP) Gel Adsorbent

**Figure 10:**
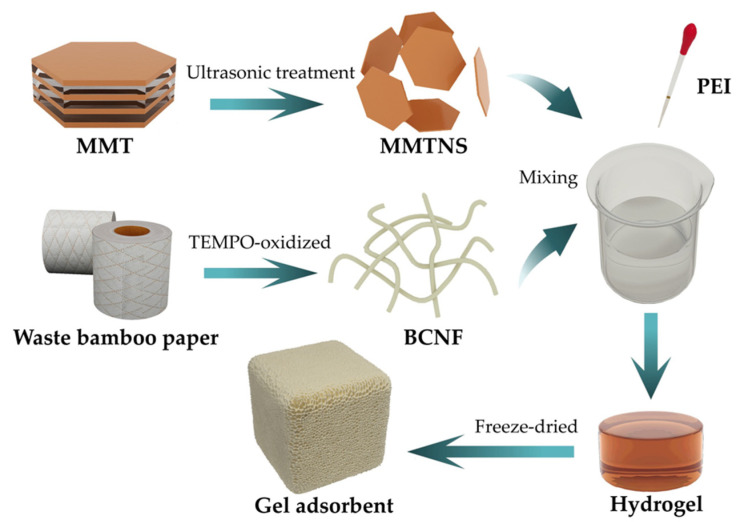
Schematic diagram of the preparation of the BMP gel adsorbent.

The preparation process is shown in Figure 10. A total of 0.6 g of BCNF and 0.6 g of MMTNS were evenly dispersed in 100 mL of deionized water by an ultrasonic treatment. Then, PEI was added to produce the hydrogel. Finally, it was pre-frozen at −20 °C and then freeze-dried at −50 °C for 72 h to obtain the resulting BMP gel adsorbent.

### 4.5. Adsorbent Characterization

Fourier transform infrared spectroscopy (FTIR) (Nicolet iS50, Thermo Fisher, Waltham, MA, USA) was carried out to detect the change of chemical groups among samples. The field emission scanning electron microscopy (SEM, SU 8010, Hitachi, Japan) equipped with energy dispersive spectroscopy (EDS) was used to analyze the morphology and elemental presence of the BMP gel adsorbent. X-ray photoelectron spectroscopic (XPS) was conducted using the Theta Probe Angle-Resolved XPS System, Thermo Fisher Scientific (UK), with an Al Ka X-ray source. The point of zero charge (pH_pzc_) of the BMP gel was determined according to the pH drift method [60]. The porosity of the BMP gel adsorbent was calculated by Image J.

### 4.6. Adsorption Experiment

The resulting gel adsorbent was applied to remove MB and Cu(II) in the batch adsorption experiment. Briefly, a 30 mg sample was added to a 30 mL adsorbate solution. The mixture was shaken at 170 rpm with a mechanical shaker for 24 h. The effects of the pH solution (MB: 2–10, Cu(II): 1–5), contact time (0–24 h), initial concentration (MB: 5–800 mg/L, Cu(II): 20–600 mg/L), and temperature (25–45 °C) on the adsorption efficiency were studied. The adsorption capacity of gel adsorbent on MB was estimated by calculating the change between the initial and residual MB concentrations using a UV spectrophotometer (UV-4802H, Unico, Shanghai, China) at 664 nm. The concentration of Cu(II) was determined using an AA-6300 atomic absorption spectrophotometer with an air acetylene burner (AAS, Shimadzu, Japan). The adsorption capacity (*q*) and removal efficiency (*R*) were calculated by Equations (1) and (2), respectively.

(1) $$q=\frac{C_{0}-C_{e}}{m}\times V$$

(2) $$R(\%)=\frac{C_{0}-C_{e}}{C_{0}}\times 100\%$$

where *q* represents the adsorption capacity (mg/g); *C*_0_ and *C_e_* are the initial concentration and equilibrium concentrations (mg/L) of the MB or Cu(II) solution, respectively; *V* represents the volume of the adsorbent solution (L); *m* is the weight of the dried adsorbent (g).

The pseudo-first-order in Equation (3), pseudo-second-order in Equation (4), and fractal-like pseudo-second-order model in Equation (5) were fitted to analyze the adsorption process.

(3) $$q_{t}=q_{e}(1-e^{-k_{1}t})$$

(4) $$q_{t}=\frac{k_{2}q_{e}{}^{2}t}{1+k_{2}q_{e}t}$$

(5) $$q_{t}=\frac{kq_{e}{}^{2}t^{a}}{1+kq_{e}t^{a}}$$

where *t* is the contact time (h); *q_e_* and *q_t_* are the adsorption capacities at equilibrium and time *t*, respectively (mg/g); *k*, *k*_1_, and *k*_2_ are the rate constants (g/(mg·h)); and *a* is the fractal exponent.

Langmuir adsorption isotherm (Equation (6)), Freundlich isotherm (Equation (7)), and the Sips isotherm model (Equation (8)) were used to analyze the adsorption isotherm.

(6) $$q_{e}=\frac{q_{m}K_{L}C_{e}}{1+K_{L}C_{e}}$$

(7) $$q_{e}=K_{F}C_{e}{}^{1/n}$$

(8) $$q_{e}=\frac{q_{m}K_{S}C_{e}{}^{1/n}}{1+K_{S}C_{e}{}^{1/n}}$$

where *C_e_* is the concentration of MB or Cu(II) at equilibrium (mg/L); *q_e_* and *q_m_* are the adsorption capacity of the adsorbent at equilibrium and the maximum adsorption capacity at saturation (mg/g), respectively; *n* is the exponent (dimensionless); *K_F_*, *K_L_*, and *K_S_* represent Freundlich, Langmuir, and Sips adsorption constants.

The separation factor (*R_L_*) was used to describe the favorable degree of the adsorption process using Equation (9):

(9) $$R_{L}=\frac{1}{1+K_{L}\times C_{0}}$$

where *R_L_* is a dimensionless equilibrium parameter or the separation factor; C_0_ is the initial pollutant concentration (mg/L); *R_L_* > 1 indicates unfavorable adsorption; *R_L_* = 1 corresponds to a linear adsorption process; 0 < *R_L_* < 1 indicates favorable adsorption, and *R_L_* = 0 means irreversible adsorption.

To investigate the thermodynamic adsorption behaviors of MB and Cu(II), the thermodynamic parameters (Δ*G°*, Δ*H°*, and Δ*S°*) were obtained using Equations (10)–(12):

(10) ΔG°=ΔH°−TΔS°

(11) $$K_{c}=\frac{q_{e}}{C_{e}}$$

(12) $$lnK_{c}=\frac{\Delta S{}^{\circ}}{R}-\frac{\Delta H{}^{\circ}}{R}\times \frac{1}{T}$$

where Δ*G°* is the Gibbs free energy change (kJ/mol); Δ*H°* is the enthalpy change (kJ/mol) and Δ*S°* is the entropy change (kJ/mol); *K_c_* is the distribution coefficient; *R* is the universal gas constant (8.314 J/mol K); *T* is the absolute temperature (*K*).

### 4.7. Reusability Experiment

A total of 30 mg of the BMP gel adsorbent was added to the 30 mL MB (100 mg/L) and Cu(II) solution (60 mg/L) for 24 h. Then, the adsorbed adsorbent was washed for 24 h with ethanol and the NaOH solution for MB and Cu(II), respectively, followed by freeze-drying at −50 °C for the next cycle. It was repeated 5 times to study the reusability.

## Figures and Tables

**Table 1:** Kinetic and isothermal parameters for adsorption of MB and Cu(II).

Model	Parameters	Adsorbate	
		MB	Cu(II)
Pseudo-first-order	*q_e_* (mg/g)	85.3679	54.593
	*k*_1_ (g/(mg·h))	6.0452	9.9634
	*R*_1_^2^	0.9024	0.7876
	*Reduced Chi-Sqr*	37.1686	17.3686
Pseudo-second-order	*q_e_* (mg/g)	91.0366	56.5531
	*k*_2_ (g/(mg·h))	0.0923	0.2877
	*R*_2_^2^	0.989	0.9386
	*Reduced Chi-Sqr*	4.1878	5.0132
Fractal-like pseudo-second-order	*q_e_* (mg/g)	94.8239	60.043
	*k* (g/(mg·h))	0.0548	0.0961
	*a*	0.7888	0.5773
	*R*_3_^2^	0.9983	0.978
	*Reduced Chi-Sqr*	0.7763	2.0980
Langmuir	*q_m_* (mg/g)	173.3430	83.8704
	*K_L_* (L/mg)	0.1935	0.0645
	*R_4_* ^2^	0.8954	0.8824
	*R_L_* (mg/L)	0 < *R_L_* < 1	0 < *R_L_* < 1
	*Reduced Chi-Sqr*	684.1315	70.1838
Freundlich	*n*	5.3604	5.3692
	*K_F_* (mg/g)/(L/mg)	59.0696	27.2935
	*R*_5_^2^	0.9637	0.9326
	*Reduced Chi-Sqr*	235.3060	40.2597
Sips	*q_m_* (mg/g)	361.8749	254.6286
	*K_S_* (L/mg)	0.2042	0.1118
	*n*	3.8214	4.0920
	*R*_6_^2^	0.9683	0.9345
	*Reduced Chi-Sqr*	239.8241	44.7173

**Table 2:** Comparison of the maximum removal capacities of MB and Cu(II) with other biosorbents.

Adsorbent	Adsorbate	pH	T (°C)	Q_max_ (mg/g)	Ref.
Graphene oxide/CNF aerogel	MB	-	-	111.2	[70]
PVA/chitosan/MMT hydrogel	MB	8	30	132.2	[71]
Cellulose-derived carbon/MMT	MB	8	25	138.1	[50]
Sugarcane bagasse	MB	-	45	9.41	[53]
BMP gel adsorbent	MB	10	25	361.9	This work
CNF aerogel	Cu(II)	6	29.85	30.0	[14]
Cellulose/acrylonitrile/methacrylic acid	Cu(II)	5.5	25	76.8	[64]
TEMPO-oxidized CNF	Cu(II)	5	30	52.3	[66]
BMP gel adsorbent	Cu(II)	5	25	254.6	This work

**Table 3:** Thermodynamic parameters of the adsorption of MB and Cu(II).

Adsorbate	*T* (K)	*K_c_*	Δ*G*° (kJ/mol)	Δ*H*° (kJ/mol)	Δ*S*° (Jmol/K)
MB	298	1.9332	−1.6331	14.8247	6.6408
	303	2.1144	−1.8863		
	308	2.3684	−2.2079		
	313	2.5517	−2.4377		
	318	2.8165	−2.7377		
Cu(II)	298	1.4213	−0.8711	8.7197	3.8453
	303	1.4331	−0.9065		
	308	1.5145	−1.0628		
	313	1.6681	−1.3315		
	318	1.7390	−1.4628		

## Data Availability

Not applicable.
